# A Novel Rapid DNA Microarray Assay Enables Identification of 37 Mycoplasma Species and Highlights Multiple Mycoplasma Infections

**DOI:** 10.1371/journal.pone.0033237

**Published:** 2012-03-29

**Authors:** Christiane Schnee, Samuel Schulsse, Helmut Hotzel, Roger D. Ayling, Robin A. J. Nicholas, Evelyn Schubert, Martin Heller, Ralf Ehricht, Konrad Sachse

**Affiliations:** 1 Institute of Molecular Pathogenesis, Friedrich-Loeffler-lnstitut (Federal Research Institute for Animal Health), Jena, Germany; 2 Institute of Bacterial Infections and Zoonoses, Friedrich-Loeffler-lnstitut (Federal Research Institute for Animal Health), Jena, Germany; 3 Department of Bacteriology, Animal Health and Veterinary Laboratories Agency Mycoplasma Group, Addlestone, United Kingdom; 4 Alere Technologies GmbH, Jena, Germany; Naval Research Laboratory, United States of America

## Abstract

Mycoplasmas comprise a conglomerate of pathogens and commensals occurring in humans and animals. The genus *Mycoplasma* alone contains more than 120 species at present, and new members are continuously being discovered. Therefore, it seems promising to use a single highly parallel detection assay rather than develop separate tests for each individual species. In this study, we have designed a DNA microarray carrying 70 oligonucleotide probes derived from the 23S rRNA gene and 86 probes from the *tuf* gene target regions. Following a PCR amplification and biotinylation step, hybridization on the array was shown to specifically identify 31 *Mycoplasma* spp., as well as 3 *Acholeplasma* spp. and 3 *Ureaplasma* spp. Members of the Mycoplasma mycoides cluster can be recognized at subgroup level. This procedure enables parallel detection of *Mollicutes* spp. occurring in humans, animals or cell culture, from mono- and multiple infections, in a single run. The main advantages of the microarray assay include ease of operation, rapidity, high information content, and affordability. The new test's analytical sensitivity is equivalent to that of real-time PCR and allows examination of field samples without the need for culture. When 60 field samples from ruminants and birds previously analyzed by denaturing-gradient gel electrophoresis (DGGE) were tested by the microarray assay both tests identified the same agent in 98.3% of the cases. Notably, microarray testing revealed an unexpectedly high proportion (35%) of multiple mycoplasma infections, i.e., substantially more than DGGE (15%). Two of the samples were found to contain four different *Mycoplasma* spp. This phenomenon deserves more attention, particularly its implications for epidemiology and treatment.

## Introduction

The genus *Mycoplasma*, one of the major taxa in the class *Mollicutes*, currently comprises more than 120 species [1]. These bacteria, which are regarded as the smallest self-replicating organisms, have unique characteristics including reduced genome size, lack of a rigid cell wall and limited number of functional metabolic pathways. Therefore, mycoplasmas have been considered models of minimal cells [2]. However, despite their apparent simplicity, several *Mycoplasma* species are significant pathogens. In humans, for instance, atypical pneumonia is associated with *Mycoplasma (M.) pneumoniae*, and genital disorders with *M. genitalium* and *Ureaplasma (U.) urealyticum*. Four mycoplasmoses are included in the list of notifiable diseases of the World Organisation for Animal Health (OIE), i.e. contagious bovine pleuropneumonia (CBPP) with the causative agent *M. mycoides* subsp. *mycoides* (formerly Small Colony type), contagious caprine pleuropneumonia (*M. capricolum* subsp. *capripneumoniae*), contagious agalactia (*M. agalactiae*), and avian mycoplasmosis (*M. gallisepticum*, *M. synoviae*). Other economically important diseases include respiratory and mammary infections of cattle caused by *M. bovis*, ocular and respiratory infection in small ruminants caused by *M. conjunctivae* or *M. ovipneumoniae*, respectively, as well as enzootic pneumonia (*M. hyopneumoniae*), arthritis and polyserositis (*M. hyorhinis, M. hyosynoviae*) in swine.

Mycoplasma contamination of cell culture is a major concern to researchers and pharmaceutical companies [3], because the unwanted presence of *M. arginini, M. hyorhinis, Acholeplasma [A.] laidlawii, M. orale, or M. fermentans* can distort the results of in vitro tests [4].

Although studies addressing dissemination and transmission pathways of the above-mentioned pathogens have been conducted [5], [6], [7], [8], [9], [10], [11], [12], [13], data on the current epidemiological situation is absent or, at best, available for selected regions only. This is partly due to the general difficulties in mycoplasma diagnosis resulting from their slow growth and requirement of fastidious culture media, as well as limitations of available tests in terms of sensitivity and specificity.

Epidemiological and clinical studies in a given animal species usually considered only the main and best characterized mycoplasmal agent, while disregarding minor or aberrant mollicutes. For instance, most projects on mycoplasmosis in cattle focused on *M. bovis* or *M. mycoides* subsp. *mycoides*, thus ignoring the possible presence of related species, such as *M. bovigenitalium*, *M. bovirhinis*, *M. bovoculi*, *M. californicum*, *M. canadense*, *M. dispar*, *M. leachii* and others. In poultry, *M. gallisepticum* is the most prominent pathogen, but *M. synoviae*, *M. iowae* and *M. imitans* should be considered as well. Likewise, small ruminants can harbor a variety of mycoplasmas besides *M. agalactiae*, e.g. members of the *Mycoplasma mycoides* cluster, *M. ovipneumoniae*, and *M. conjunctivae*. Diagnostic evidence from recent years clearly suggests that host specificity of animal mycoplasmas is generally not stringent [14], but more comprehensive investigations are required.

Very little is known about the frequency of co-infection by two or more mycoplasmal agents [15], [16], both at single-animal and herd levels, thus preventing proper assessment of the synergetic and/or competitive effects involved. The diagnostic challenges resulting from the multitude of mycoplasma organisms potentially involved can be efficiently addressed only if adequate detection methods are available. A single PCR or ELISA test would not necessarily identify atypical or co-infecting agents present in a sample. While PCR combined with denaturing-gradient gel electrophoresis (DGGE) can detect mixed infections, it is laborious and complex to perform and interpret [17].

In contrast, DNA microarray testing opens up new possibilities for laboratory diagnosis. The possibility of using a large number of detection probes covering discriminatory gene segments and/or multiple genomic regions of many different microbial agents confers a high degree of parallelity to this technology. Therefore, DNA microarray assays can attain far higher diagnostic resolution than PCR. The broad use of array technology in rapid diagnosis of bacterial and viral pathogens, however, is only emerging.

In the present study, we developed a rapid DNA microarray assay capable of identifying at least 37 *Mollicutes* spp., among them important human and animal pathogens and cell culture contaminants.

## Materials and Methods

### Mycoplasma strains

The type or reference strains used are listed in Table 1. Field strains were from the collection of the National Reference Laboratory for CBPP (Head: MH). Culture was conducted according to standard methodology [18].

**Table 1:** Summary of hybridization test results of 44 *Mollicutes* organisms on two genomic target sites.

Species/Taxon	Type strain	Specific detn. 23S rDNA	Specific detn. *tuf* gene	Field strains tested	Comment
*A. axanthum*	S743	+	+	2	
*A. laidlawii*	PG8	+	+	5	
*A. modicum*	PG49	+	+	2	
*M. adleri*	G145	+	n.d.	0	
*M. agalactiae*	PG2	+	+*	3	* *M. bovis*
*M. alkalescens*	PG51	+	+	5	
*M. alvi*	ILSLEY	+	+	3	
*M. arginini*	G230	+*	+*	3	* *M.gateae*
*M. bovigenitalium*	PG11	+	+	3	
*M. bovirhinis*	PG43	+	+	6	
*M. bovis*	PG45	+	+*	24	* *M. agalactiae*
*M. bovoculi*	M165/69	+	+	2	
*M. californicum*	ST-6	n.d.	+	2	
*M. canadense*	275C	n.d.	+	3	
*M. canis*	PG14	+	+	4	
*M. capricolum* subsp. *capricolum*	California Kid	Mmyc. cluster	Mmyc. cluster	2	
*M. capricolum* subsp. *capripneumoniae*	F38	Mmyc. cluster	Mmyc. cluster	2	
*M. conjunctivae*	HRC581	+	+	4	
*M. dispar*	462/2	+	+	4^#^	^#^mixed culture
*M. fermentans*	PG18	+	+	2	
*M. gallinarum*	PG16	+	+	3	
*M. gallisepticum*	PG31	+*	+	6	* *M.imitans*
*M. gateae*	CS	n.d.	+*	0	* *M.arginini*
*M. genitalium*	G37	+*	+	0	* *M.pneumoniae*
*M. hominis*	PG21	+	+	6	
*M. hyopneumoniae*	J	+	+	1	
*M. hyorhinis*	BTS-7	n.d.	+	4	
*M. imitans*	4229	+	+	0	
*M. iowae*	695	+	+	6	
*M. leachii*	PG50	Mmyc. cluster	Mmyc. cluster	2	
*M. meleagridis*	N17529	+	+	6	
*M. mycoides* subsp. *capri*	PG3	Mmyc. cluster	Mmyc. cluster	2	
*M. mycoides* subsp. *mycoides* (SC)	PG1	Mmyc. cluster	Mmyc. cluster	2	
*M. orale*	CH19299	+	+	3	
*M. ovipneumoniae*	Y98	+	+	7	
*M. pneumoniae*	FH	+*	+	1	* *M.genitalium*
*M. pulmonis*	Ash/PG34	+	+	1	
*M. putrefaciens*	KS-1	+	+	2	
*M. salivarium*	PG20	+	+	0	
*M. synoviae*	WVU1853	+	+	3	
*M. verecundum*	107	+	+	0	
*U. diversum*	A417/C (NCTC10182)	n.d.	+	2	
*U. parvum*	27	+	+*	2	* *U.urealyticum*
*U. urealyticum*	960	+	+*	2	* *U.parvum*

* cross-reaction with related species, n.d. not done (no specific probes identified in that locus).

### Field samples

The majority of the field samples tested originated from investigations by the AHVLA Regional Laboratories in England and Wales between 2009 and 2011, where disease investigations and, in some cases, post-mortems had been performed. Clinical samples as detailed in Table 2 and File S1 were submitted to the Mycoplasma Group (AHVLA, Weybridge, UK) in Eaton's media [19]. DNA was extracted directly from the sample using a Maxwell 16 automated system and Maxwell tissue DNA purification kit (Promega, Southampton, UK) and stored at −20°C until testing. Other samples included in Table 2, were sample references: 14F11, 20F11, 22F11, 23F11, 33F11, and 39F11, which were submitted as freeze-dried clinical samples from Iran for identification by the Mycoplasma Group as they are the OIE Contagious Agalactia Reference Laboratory (Head: RN). Sample SR00 came from a culture collection, and samples 82A10 and 83A10 were submitted as freeze-dried culture samples as part of a proficiency test.

**Table 2:** Summary of test results of DNA microarray and DGGE assays on 51 clinical tissue samples and 9 cultures from field samples.

Sample ID	Sample type	DGGE	DNA microarray	Comment
100SR10	ovine, lung	*M. arginini, M. ovipneumoniae*	*M. arginini* 3, *M. ovipneumoniae*	concordant, dual infection
34 B 10	bovine, lung	*M. bovis, M. alkalescens*	*M. bovis, M. alkalescens*	concordant, dual infection
108 B 10	bovine, lung	*M. bovis, M. alkalescens* 1	*M. bovis, M. alkalescens* 1	concordant, dual infection
13SR11	ovine, nasal swab	*M. ovipneumoniae, M. arginini*	*M. ovipneumoniae, M. arginini* 3	concordant, dual infection
33F11	culture, ovine, milk	*M. agalactiae*	*M. agalactiae, M. putrefaciens*	more species by AS
19B10	bovine, lung	*M. alkalescens, M. bovis*	*M. alkalescens, M. bovis, M. dispar* 2	more species by AS
490 B 09	bovine, vaginal swab	*M. bovigenitalium*	*M. bovigenitalium* 3, *M. alkalescens* 1	more species by AS
485 B 09	bovine, vaginal swab	*M. bovigenitalium*	*M. bovigenitalium, M. alkalescens* 1	more species by AS
53B10	bovine, swab	*M. bovirhinis*	*M. bovirhinis, M. bovis* 1, *M. arginini* 2 ^*,*^ 3	more species by AS
365B10	bovine, nasal swab	*M. bovirhinis, M. dispar*	*M. bovirhinis, M. dispar, M. bovis* 1, *M.arginini* 2 *^,^* 3	more species by AS
36B10	bovine, lung	*M. bovis*	*M. bovis, M. arginini* 1 *^,4^*	more species by AS
49B10	bovine, lung	*M. bovis*	*M. bovis, M. dispar* 1	more species by AS
279B11	bovine, lung	*M. bovis*	*M. bovis, M. dispar* 1	more species by AS
669 B 09	bovine, eye swab	*M. bovoculi*	*M bovoculi, M. canadense*	more species by AS
128SR09	ovine, eye swab	*M. conjunctivae*	*M. conjunctivae, M. ovipneumoniae* 1, *M. arginini* 2	more species by AS
32 B 10	bovine, lung	*M. dispar*	*M. dispar, M. bovis* 1, *M. alkalescens* 1, *M. bovirhinis* 2	more species by AS
39 B 10	bovine, lung	*M. dispar*	*M. dispar* 1, *M. bovis* 1, *M. alkalescens* 1	more species by AS
265B10	bovine, nasal swab	*M. dispar*	*M. dispar, M. arginini* 2 *^,^* 3	more species by AS
83A10	culture from strain collection	*M. meleagridis*	*M. meleagridis, M. dispar* 2	more species by AS
120A10	chicken, eyelid	*M. synoviae*	*M. synoviae, M. iners*	more species by AS
95SR10	ovine, swab	unidentified bands	*M. conjunctivae*	AS more specific
79O10	caprine, lung	*M. arginini, M. ovipneumoniae* 2	*M arginini* 3	more species by DGGE
142 B 09	bovine, lung	*M. bovirhinis, M. alkalescens* 1	*M. bovirhinis*	more species by DGGE
89 B 10	bovine, lung	*M. bovirhinis, M. alkalescens* 1	*M. bovirhinis, M. dispar* 1	discordance in second agent
36 samples	tissue (29) and culture (7)	*M. agalactiae, M. alkalescens, M. arginini, M. bovirhinis, M. bovis, M. canadense, M. capricolum* subsp. *capricolum, M. conjunctivae, M. gallisepticum, M. mycoides* subsp. *capri, M. ovipneumoniae, M. putrefaciens, M. synoviae*	concordant, monoinfections	

1 confirmed by species-specific PCR,

2 species-specific PCR negative,

3 not distinguishable from *M. gateae*.

### DNA extraction

Cultured mycoplasma strains were DNA extracted using the High Pure PCR Template Preparation Kit (Roche Diagnostics, Mannheim, Germany) according to the instructions of the manufacturer.

### Sequencing the 23S rRNA and *tuf* genes

Partial 23S rDNA sequences of mycoplasma strains were determined. Primers F1388 (5′-GTT TCC TGG GCA AGG TTC G-3′) and R1982 (5′-CCG TTA TAG TTA CGG CCG CC-3′) were used to amplify a 600-bp segment in the central domain of the gene.

Primer pair tuf-064F (5′-ATGCCNCAAACWMGWGAACAC-3′)/tuf-681R (5′-TRTGACKWCCACCTTCWTCTT-3′) was selected from sites of highest homology in the alignment. The 614-bp central region flanked by these primers was the final target region in the *tuf* gene that was further analyzed for probe design. All sequencing was conducted by Eurofins MWG Operon (Ebersberg, Germany). New sequences determined in the present study have been deposited in the GenBank database under the following accession numbers: JQ390341–JQ390384 (23S rDNA), JQ390385–JQ390408 (*tuf* gene).

### *In silico* sequence analysis and selection of hybridization probes

An alignment of experimentally determined 23S rDNA sequences from 44 taxa listed in Table 1 was processed using the Vector NTI Advance 11 software (Invitrogen, Carlsbad, CA, USA), which is based on the ClustalW algorithm. The 471-nt alignment is provided in File S2.

A total of 19 *tuf* gene sequences of *Mollicutes* included in Table 1 were available from GenBank (18 as part of complete genomes and the CDS of *M. canis*). These sequences were combined with 24 *de novo* sequences in an alignment of the central 614-nt region (File S3). The sequence of *M. adleri* was not available at the time of array production.

Two different probe selection strategies were used. a) In the case of the 23S rDNA target region, probe binding sites were selected manually on the basis of uniqueness, i.e. each probe was designed to be specific for its eponymous mycoplasma species and checked by BLAST analysis. b) For the *tuf* target, hybridization probe design included processing of the alignment from File S3 using the program E-INS-I of the MAFFT package [20], version 6.853b (2011/04/27), which is available from http://mafft.cbrc.jp/alignment/software/. Subsequently, the in-house software package Clondiag ArrayDesign (Alere Technologies, Jena, Germany) was used to fine-tune and select the best-discriminating probes.

The following basic selection criteria were used for the hybridization probes: i) specificity of the target sequence (i.e. uniqueness, at least one nucleotide difference to second best match in the case of 23S probes), ii) melting temperature in the range from 54 to 62°C, and iii) absence of significant self-complementarity. The Oligonucleotide Properties Calculator (http://www.unc.edu/~cail/biotool/oligo/) was used to check these parameters. The selected oligonucleotides (70 for 23S rDNA; 86 for *tuf*) had an average size of 28.4 nt (min. 23/max. 34), a melting temperature of 59.0°C (54/62), and a G+C content of 40.3 mol-% (27/57).

Nucleotide sequences and basic physical parameters of all probes are provided in File S4. Each substance was spotted three-fold onto the microarray. Biotinylated oligonucleotide probes (staining controls), and spotting buffer (background control) were also included. Production of the microarrays was described previously [21].

### Pre-hybridization amplification (Biotinylation PCR)

The 5′-biotinylated primers F1388 and R1982 were used to amplify an approximately 600-bp segment containing the signature region of the 23S rRNA gene. The 620-bp *tuf* target region was amplified using 5′-biotinylated primers tuf-064F and tuf-681R. Pre-hybridization amplification reactions were run on either real-time (two simplex reactions) or conventional (duplex) protocols.

In real-time PCR, the reaction mix for each target contained 1 µl (10–100 ng) of mycoplasma chromosomal DNA, 500 nM of both forward and reverse primer, 10 µl of DyNAmo™ Flash SYBR® Green qPCR Mastermix (Finnzymes, Vantaa, Finland), and was made up to 20 µl with deionized water. After initial denaturation at 95°C for 10 min, 40 cycles (95°C for 30 s, 52°C for 30 s and 72°C for 60 s) with subsequent dissociation curve analysis were run on a Mx3000P® thermocycler and processed using the MxPro™ 4.10 software (both from Agilent, Waldbronn, Germany).

In conventional PCR experiments, both primer pairs were used in a duplex amplification protocol. Each reaction mix contained 1 µl (10–100 ng) of mycoplasma chromosomal DNA, 400 nM of each primer, 2.5 mM MgCl_2_, 1 mM dNTP mix, 2.5 µl of 10× PCR Buffer, 0.5 U of *Taq* DNA polymerase (reagents from 5 Prime, VWR, Darmstadt, Germany), and was made up to 25 µl with water. The cycling profile included initial denaturation at 95°C for 60 s, 40 cycles (95°C for 30 s, 52°C for 30 s and 72°C for 60 s) and final elongation at 72°C for 60 s on a Thermocycler T3 (Biometra, Göttingen, Germany). For inspection, products were separated on 1.5% agarose gels, stained with ethidium bromide and visualized by UV illumination.

### DNA microarray hybridization

Optimal hybridization conditions were determined empirically by varying hybridization temperatures from 50°C to 60°C and washing step temperatures immediately after hybridization from 35°C to 47°C. The Identibac Hybridisation Kit (Alere) was used according to the instructions of the manufacturer. Briefly, the AS vessels were conditioned by washing with 200 µl of deionized water and 100 µl of hybridization buffer C1 at 50°C for 5 min. All incubations were conducted upon shaking at 550 rpm on a BioShake iQ (Quantifoil Instruments Jena, Germany). One µl of the PCR product (0.5 µl of each simplex product from real-time PCR) was diluted in 99 µl Hybridization Buffer in a separate tube, heated at 95°C for 5 min and put on ice for 30 s. Once transferred into the AS, DNA reassociation was allowed at 50°C for 60 min. Supernatants were discarded and the array was washed twice with 200 µl of washing buffer C2 at 45°C for 10 min. Subsequently, 100 µl of horse radish peroxidase conjugate solution (1 µl C3 and 99 µl C4) were added to the tubes and incubated at 30°C for 10 min. The vessels were then washed with 200 µl of washing buffer C5 at 30°C for 4 min before reactive spots were finally visualized using 100 µl of Seramun Grün (D1) as peroxidase substrate. Hybridization signals were measured using the ArrayMate transmission reader (Alere).

### Processing of AS hybridization data using the PatternMatch algorithm

Hybridization signals were processed using the Iconoclust software, version 3.3 (Alere). Normalized intensities of the spots were calculated automatically by the software using the following equation: NI = 1−(M/BG) (where NI is normalized intensity, M is average intensity of the automatically recognized spot, and BG is intensity of local background). NI values would theoretically range from 0 (no signal) to 1 (maximum signal).

A global specificity table listing the number of mismatches of each probe to all mycoplasma species per target (“Probe matching matrix”, File S4) was used to construct theoretical hybridization patterns (i.e. signal intensity of 0.9 for perfect match, 0.6 for 1 mismatch, 0.3 for 2 mismatches, 0.1 for 3 mismatches, no signal for more mismatches, at medium stringency).

The assignment of hybridization patterns obtained from the 23S rDNA and *tuf* gene sectors of the array was based on probe-by-probe comparison of the measured signals of a given sample with theoretically expected signals of all reference strains. For this operation, the PatternMatch algorithm was used, which is an integral part of the Partisan ArrayLIMS database software system (Alere). The final numerical output is given as the matching score (MS), which represents the sum of differences between corresponding signal intensities of sample and reference. Thus, the MS value is a measure of dissimilarity between two hybridization patterns. An ideal match of two patterns based on the same set of oligonucleotide probes will yield MS = 0, whereas values above 40 require critical scrutiny because they may indicate a poor match or multiple infection. In the latter case, additional manual assignment is necessary. The Delta MS value, defined as the arithmetic difference between best and second best match [22], served as measure for the accuracy of mycoplasma species identification. A value higher than 0.5 was considered as sufficient for unambiguous distinction between two patterns.

### Denaturing gradient gel electrophoresis (DGGE)

DNA preparations from field tissue samples were amplified by PCR and analyzed by DGGE as described previously [17].

### Confirmatory PCR testing

Field samples giving different results in DGGE and DNA microarray assay were additionally examined using species-specific PCR protocols from the literature for *M. bovis* [23], *M. bovirhinis*, *M. alkalescens* [24], *M. dispar* [25], *M. ovipneumoniae* [26], and *M. arginini* [27].

## Results

### Analysis of the 23S ribosomal RNA gene region and probe selection

**Figure 1:**
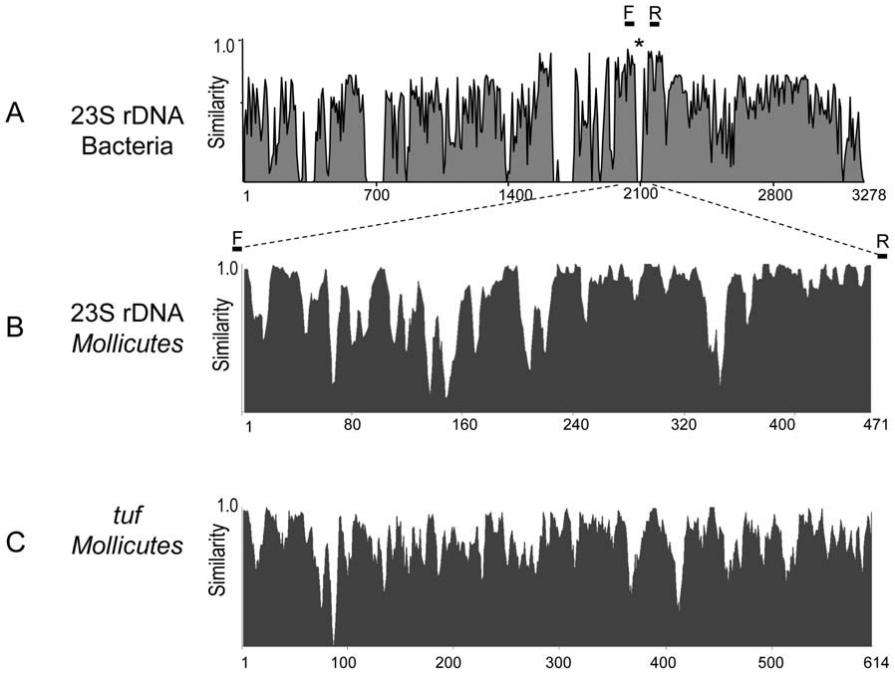
Sequence similarity plots of the target regions used in the present microarray. Numbers on the abscissa denote positions in the sequence alignment. The diagrams were produced using Vector NTI 11 and are based on alignments of A) complete 23S rRNA genes of 10 selected eubacterial species and 9 mycoplasmas (asterisk showing the location of the 23–26-nt deletion found in all *Mollicutes* spp.), B) the 471-nt signature region of all 44 mycoplasmas included in this study (alignment in File S2), and C) the central 614-nt region of the *tuf* gene of 43 mycoplasma species (alignment in File S3). Bars denoted F and R indicate the positions of forward and reverse primers, respectively, that were used for amplification. MVW 1–3 indicate the positions of most variable windows.

An alignment of 23S rDNA sequences from eubacteria and mycoplasmas revealed an alternate distribution of highly conserved and variable segments over the entire gene locus, which is illustrated in the similarity plot in Fig. 1A. Unlike the cell-walled bacterial species, all mycoplasmas examined showed a 23–26 nt deletion in the segment around position 2100. This observation prompted us to sequence type strains of 38 *Mycoplasma*, 3 *Acholeplasma* and 3 *Ureaplasma* spp. in this variable region. The similarity plot of the aligned segments from 44 *Mollicutes* spp. in Fig. 1B shows the considerable sequence diversity (alignment given in File S2). Subsequently, we systematically explored this domain for discriminatory sites. Manual selection of species-specific hybridization probe binding sites led to the definition of 107 oligonucleotide probes, of which 70 were confirmed after two rounds of specificity testing (data not shown), i.e. 64 probes for 41 species, 4 for the *Mycoplasma mycoides* cluster, as well as genus-specific probes for *Mycoplasma* (6), *Acholeplasma* (1) and *Ureaplasma* (1). It was not possible to find functional probes in this target region for the following species: *M. californicum*, *M. canadense*, *M. gateae*, *M. hominis*, *M. hyorhinis*, and *U. diversum*.

To facilitate automatic assignment of measured signals to individual species, theoretical hybridization patterns were constructed based on the expected dependence of signal intensity on the number of nucleotide mismatches between target and probe. Previous studies had shown that, depending on the level of stringency of the hybridization reaction, signal reductions caused by one, two or three mismatches were directly measurable [28], [29]. All 44 theoretical hybridization patterns from the 23S rDNA sector were placed in the database.

### Analysis of the *tuf* gene region and probe selection

To extend the discriminatory capacity of the microarray, the *tuf* gene was considered as an additional target. Nineteen gene sequences were retrieved from GenBank and combined in an alignment with *de novo* sequences of further 24 taxa from Table 1 (alignment given in File S3). As the extent and nature of sequence diversity did not allow a consistent selection of strictly species-specific probes as in the case of 23S rDNA, the objective was to define combinatorial sets of probes leading to characteristic hybridization patterns that can be assigned to individual species. The alignment of 614-nt segments of 43 *tuf* genes was processed using E-INS-I, which led to the definition of three most variable windows whose positions are indicated in Fig. 1C. Within these three windows, the software identified 86 oligonucleotide probes satisfying the basic selection criteria. All theoretically expected *tuf* patterns were entered in the database.

### Validation of the 23S rRNA and *tuf* probes

**Figure 2:**
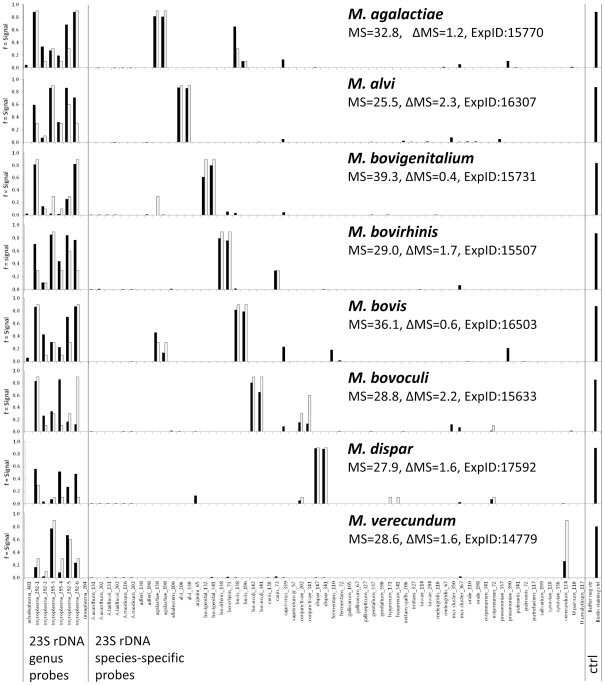
Differentiation based on 23S rDNA probes among eight *Mycoplasma* species potentially occurring in cattle. Black bars denote experimental signals, gray bars denote theoretically predicted signals. Each hybridization experiment is characterized by matching score (MS) and accuracy (Delta MS, see Materials and Methods). Control bars at the right-hand margin show spotting buffer (background control) and biotinylated oligonucleotide (staining control).

A total of 44 type strains of the *Mollicutes* organisms were examined using the present DNA microarray assay protocol. In addition, 129 field strains from 37 species were examined, whose identity had been previously established using DGGE, DNA sequencing or immunofluorescence. The results are summarized in Table 1. In the case of 27 species, the 23S rDNA probe panel gave rise to theoretically expected species-specific hybridization patterns consisting of genus and species probe signals only. The discriminatory potential of this target region is illustrated in Fig. 2, where the distinction of eight different bovine mycoplasmas based on specific probes for genus and species is shown.

However, 23S rDNA probes alone did not allow the identification of all organisms on the list. In addition to the six species lacking specific probes (see above), four species could not be unambiguously recognized by their probe signals, i.e. *M. arginini*, *M. gallisepticum*, *M. genitalium*, and *M. pneumoniae*, because of cross-reactions with closely related species. Furthermore, *M. mycoides* with its subspecies *mycoides* and *capri*, *M. capricolum* with its subspecies *capricolum* and *capripneumoniae*, as well as *M. leachii* could be identified as members of the Mycoplasma mycoides cluster, and differentiation between mycoides and capricolum subgroups was possible.

**Figure 3:**
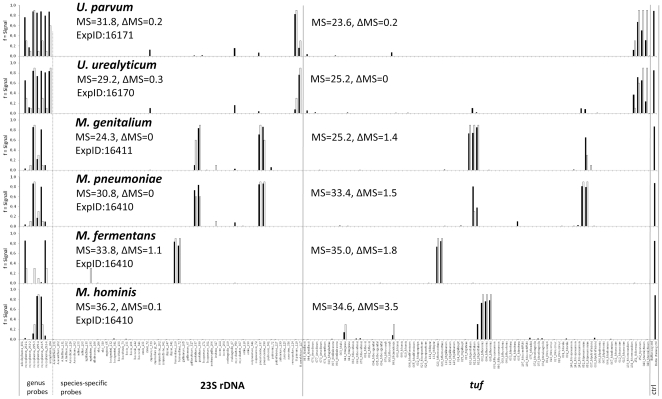
Differentiation among human *Mycoplasma* and *Ureaplasma* spp. based on the combination of probes from 23S rDNA and the *tuf* gene. Matching scores (MS) and Delta MS values are given for both gene loci in each hybridization experiment. Black bars denote experimental signals, gray bars denote theoretically predicted signals. Control bars at the right-hand margin show spotting buffer (background control) and biotinylated oligonucleotide (staining control).

The *tuf* gene segment offered additional discriminatory capacity. It can be seen from the test results in Table 1 that 34 species have been unambiguously identified by probes from this target. The sister species *M. pneumoniae* and *M. genitalium*, both relevant human pathogens, show identical hybridization patterns each for the 23S rDNA target, but are well distinguishable by their specific hybridization pattern on *tuf* probes (Fig. 3). Cell culture contaminants, such as *A. laidlawii*, *M. arginini*, *M. hyorhinis*, and *M. orale*, can also be readily identified. Conversely, the closely related pairs of *M. bovis/M. agalactiae* (Fig. 2) and *U. urealyticum/U. parvum* showed very similar patterns on *tuf* (data not shown), whereas the signals from 23S rDNA probes were straightforward and discriminatory.

When combining the results from both target genes, a total of 37 type strains were correctly assigned at species level. Similar to the findings from the 23S rDNA site, the *tuf* probes failed to completely differentiate among members of the *Mycoplasma mycoides* cluster, but the two subgroups of the cluster could be differentiated as above. The high sequence homology did not allow the selection of discriminatory probes for each of the five members. The remaining critical pairs include the closely related *M arginini* and *M. gateae* from the *M. hominis* cluster, which were not distinguishable using the present set of probes.

### Analytical sensitivity

The sensitivity of the combined PCR-microarray assay was evaluated by examining decimal dilutions of spectrophotometrically quantified genomic DNA (100 pg to 1 fg) from the type strains of *M. bovis* and *M. dispar*. When these mycoplasmas were analyzed separately, 100 fg of DNA corresponding to approximately 100 genome copies were found to be sufficient to obtain species-specific hybridization patterns. To assess the technique's capability to detect co-infections, the two test DNAs were mixed at different ratios, co-amplified by SYBR Green real-time PCR and hybridized on ArrayStrips. The presence of a 10^3^-fold excess of *M. bovis* DNA did not result in a deteriorated detection limit for *M. dispar* (data not shown). This illustrates the usability of the test for parallel and simultaneous detection of multiple mycoplasma species with differing loads in a clinical sample.

### Testing of field samples

A panel of 60 samples (31 from cattle, 25 from small ruminants and 4 from birds) previously examined by DGGE was tested blind on the present microarray. The results in Table 2 show that concordant results were obtained in the majority of cases. Mycoplasma organisms identified by DGGE were confirmed by the microarray test in 59 (98.3%) instances, among them 36 samples with a mycoplasma monoinfection. One sample with an unclear DGGE result was unambiguously identified as *M. conjunctivae*.

**Figure 4:**
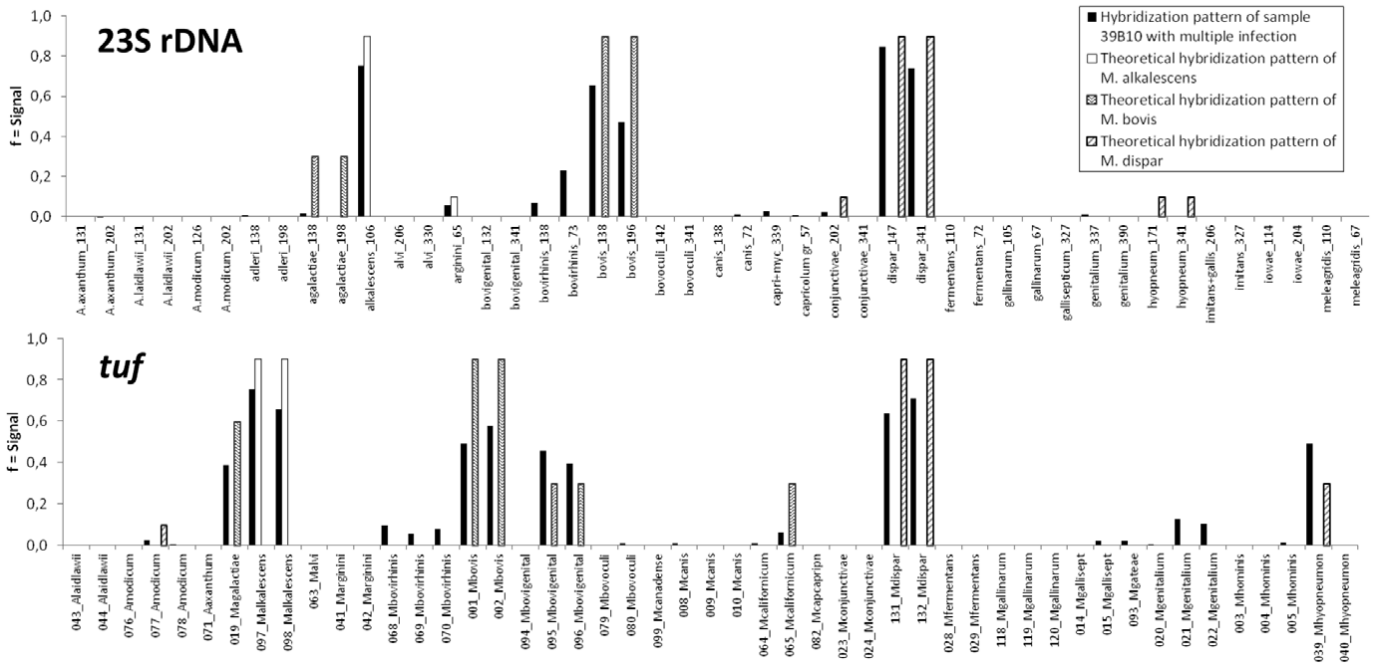
Detection of multiple mycoplasma infection in a DNA extract from bovine lung tissue (sample 39 B 10, **Table 2**). The diagram shows the combined pattern match of sample and three matching *Mycoplasma* spp. Black bars denote the hybridization signals of the sample, while theoretically predicted signals for *M. alkalescens*, *M. bovis* and *M. dispar* are represented by empty, dashed and dotted bars, respectively. This close-up presentation shows only the relevant sections of the diagram.

Notably, the microarray revealed a large proportion of samples containing multiple mycoplasma infection (21/60 = 35.0%) compared with DGGE (9/60 = 15.0%). There were identical results in both tests for 4 samples harboring two different species. The microarray test detected additional mycoplasmas in 16 cases (10 confirmed by independent test), and DGGE in 2 cases (1 confirmed). In 6 samples, the microarray identified more than two mycoplasma species (3 confirmed). As an example, detection of *M. alkalescens*, *M. bovis* and *M. dispar* in a bovine lung tissue sample is shown in Fig. 4.

## Discussion

DNA microarray assays using the present platform have already been used for a number of microbial pathogens [21], [30], [31]. For the detection of mycoplasmas, Volokhov et al. [32] described an alternative array technology. The paper featured a slide-based microarray system with probes derived from the 16S–23S intergenic transcribed spacer region and involved fluorescence labeling of targets. The system was shown to identify cultured strains of 24 *Mollicutes* spp. The novelty of the present approach consists in the combination of two basically different probe sets for differentiation among related species, i) the 23S rRNA gene probes picked manually in similarity minima of the signature region, and ii) the combinatorial set of probes derived from the central region of the *tuf* gene. While the former probe set allows direct identification according to the hybridization signals of the species-specific probes (yes/no decision), the latter was designed to produce hybridization patterns that can be assigned to individual species. The use of different probe design strategies resulted from the observed differences in inter-species sequence diversity within the 23S ribosomal and *tuf* gene loci. Similarity plots in Fig. 1 show the higher abundance of low-similarity sites in the ribosomal target, all of which represent potential binding sites for highly discriminatory probes.

While the present analysis of the 23S rRNA gene region in *Mollicutes* for diagnostic purposes is new, the remarkable potential of the gene encoding elongation factor Tu was recognized more than a decade ago. Kamla and co-workers [33] already showed that it represented a better phylogenetic marker than the 16S rRNA locus. Later on the *tuf* gene was used as target in a broad-range real-time PCR assay for detection of a group of mycoplasma species [34]. In the present study, we took advantage of the locus' discriminatory potential for *Mollicutes* through the combinatorial approach. This potential can be further exploited in future studies as it will allow the addition of probes for more mycoplasma species of interest on an extended version of the microarray. Altogether, the two-target approach led to an increase of the microarray assay's discriminatory capacity, as well as an improvement of the accuracy of species identification.

The findings of the present study demonstrate the excellent diagnostic potential of the microarray-based methodology. For instance, the possibility of running a single test to monitor all mycoplasmas in human samples (Fig. 3) can be a promising time-saving and economical alternative. The same applies to testing for cell culture contaminants. In veterinary diagnosis, simultaneous detection of different mycoplasma agents occurring in cattle (Fig. 2), in small ruminants including *M. agalactiae*, *M. conjunctiviae*, *M. ovipneumoniae*, mycoides cluster members, or in poultry including *M. gallisepticum*, *M. meleagridis*, *M. synoviae*, *M. iowae*, *M. imitans*, renders the setup of individual tests for each agent unnecessary.

Furthermore, the present assay is an efficient tool to investigate dual and multiple mycoplasma infections in individual animals. The present panel of samples was found to contain an unexpectedly high proportion of these infections, i.e. 35%. Although we cannot rule out that the panel has a bias towards multiple infections, the findings indicate that the simultaneous presence of different *Mycoplasma* spp. is no rare event. In addition, the present data is raising intriguing questions on interactions and synergies between individual microorganisms, as well as their consequences for epidemiology and therapy, which have to be addressed in future studies.

Differentiation among members of the *Mycoplasma mycoides* cluster remains a particularly difficult problem. Even after the recent revision of its taxonomy [35], the remaining five member organisms are still closely related. In addition, intra-taxon heterogeneity is poorly investigated, but probably not negligible. Unambiguous identification of all cluster members based on combined 23S rDNA and *tuf* gene targets was not possible.

Direct identification of pathogens from clinical samples is an important asset of the ArrayStrip assay. In accordance with our previous finding that the sensitivity of the present DNA microarray platform was equivalent to that of real-time PCR [36], examination of 60 field samples (Table 2) confirmed that a valid hybridization pattern was obtained as soon as a sample contained sufficient DNA template to yield a PCR amplicon. In this context, the microarray test can supersede time-consuming culture experiments and, in the absence of a clear idea about the identity of the pathogen in the sample, avoid the necessity of running several different tests.

The high specificity of the present assay results from the large number of oligonucleotide probes on the array, which simultaneously interrogate the sample DNA during hybridization. For each mycoplasma organism, there are multiple probes, i.e. one to three from the 23S rDNA and a combinatorial set from the *tuf* gene.

Alternative approaches to parallel detection tests include melting curve analysis [37] and bead-based Luminex assays [38], [39]. However, while rapid, highly specific and reasonably sensitive, the parallelity of these technologies is practically limited to about ten different species in a single test.

All in all, the ArrayStrip microarray test is suitable for routine diagnosis as shown for other pathogens [30], [40], [41], mainly for its ease of operation, rapidity, potential to high throughput, high information content, and affordability. The major steps include DNA extraction using a commercial kit, amplification by duplex biotinylation PCR, as well as hybridization, washing and staining, and results are available within a working day. Apart from the ArrayStrips and the transmission reader, the assay requires only standard laboratory equipment. The fact that the final output is based on automatic comparison of measured hybridization signals with reference patterns in the database adds a reasonable degree of objectivity.

We conclude that we have developed a promising diagnostic tool for rapid detection of mono- and multiple infections of 42 *Mollicutes* spp., including subgroup identification of Mycoplasma mycoides cluster members. The combination of species-specific and combinatorial probes, which has facilitated differentiation between closely related species, can be further extended in this open system to include additional organisms of interest. The present DNA microarray assay can be used in diagnosis of human and animal infections, as well as cell culture contamination.

## Supporting Information

File S1 **Complete list of field samples and individual test results of DGGE and DNA microarray testing**
(XLS)

File S2 **Alignment of 23S rDNA sequences**
(MSF)

File S3 **Alignment of** ***tuf*** **sequences**
(MSF)

File S4 **List of oligonucleotide probes on the array and Probe Matching Matrix**
(XLS)
